# Exploring autism spectrum disorder and co-occurring trait associations to elucidate multivariate genetic mechanisms and insights

**DOI:** 10.1186/s12888-024-06392-w

**Published:** 2024-12-18

**Authors:** Karoliina Salenius, Niina Väljä, Sini Thusberg, Francois Iris, Christine Ladd-Acosta, Christophe Roos, Matti Nykter, Alessio Fasano, Reija Autio, Jake Lin, the GEMMA study

**Affiliations:** 1 Faculty of Medicine and Health Technology, Tampere University and Tays Cancer Centre, Tampere, Finland; 2 The GEMMA study, GEMMA-PROJECT.eu, Salerno, Italy; 3 BMSystems, Paris, France; 4 Department of Epidemiology, Johns Hopkins Bloomberg School of Public Health, Baltimore, USA; 5 Euformatics, Tekniikantie, Espoo, Finland; 6 Foundation for the Finnish Cancer Institute, Helsinki, Finland; 7 European Biomedical Research Institute of Salerno (EBRIS), Salerno, Italy; 8 Harvard Medical School, Harvard T.H. Chan School of Public Health, Boston, USA; 9 Health Sciences, Faculty of Social Sciences, Tampere University, Tampere, Finland; 10 Department of Medical Epidemiology and Biostatistics, Karolinska Institute, Stockholm, Sweden

**Keywords:** ASD, ASD genetically correlated traits, Multivariate GWAS, Mendelian randomization, GEMMA

## Abstract

**Background:**

Autism spectrum disorder (ASD) is a partially heritable neurodevelopmental trait, and people with ASD may also have other co-occurring trait such as ADHD, anxiety disorders, depression, mental health issues, learning difficulty, physical health traits and communication challenges. The concomitant development of ASD and other neurological traits is assumed to result from a complex interplay between genetics and the environment. However, only a limited number of studies have performed multivariate genome-wide association studies (GWAS) for ASD.

**Methods:**

We conducted to-date the largest multivariate GWAS on ASD and 8 ASD co-occurring traits (ADHD, ADHD childhood, anxiety stress (ASDR), bipolar (BIP), disruptive behaviour (DBD), educational attainment (EA), major depression, and schizophrenia (SCZ)) using summary statistics from leading studies. Multivariate associations and central traits were further identified. Subsequently, colocalization and Mendelian randomization (MR) analysis were performed on the associations identified with the central traits containing ASD. To further validate our findings, pathway and quantified trait loci (QTL) resources as well as independent datasets consisting of 112 (45 probands) whole genome sequence data from the GEMMA project were utilized.

**Results:**

Multivariate GWAS resulted in 637 significant associations (*p* < 5e-8), among which 322 are reported for the first time for any trait. 37 SNPs were identified to contain ASD and one or more traits in their central trait set, including variants mapped to known SFARI ASD genes *MAPT*,* CADPS* and *NEGR1* as well as novel ASD genes *KANSL1*, *NSF* and *NTM*, associated with immune response, synaptic transmission, and neurite growth respectively. Mendelian randomization analyses found that genetic liability for ADHD childhood, ASRD and DBT has causal effects on the risk of ASD while genetic liability for ASD has causal effects on the risk of ADHD, ADHD childhood, BIP, WA, MDD and SCZ. Frequency differences of SNPs found in *NTM* and *CADPS* genes, respectively associated with neurite growth and neural/endocrine calcium regulation, were found between GEMMA ASD probands and controls. Pathway, QTL and cell type enrichment implicated microbiome, enteric inflammation, and central nervous system enrichments.

**Conclusions:**

Our study, combining multivariate GWAS with systematic decomposition, identified novel genetic associations related to ASD and ASD co-occurring driver traits. Statistical tests were applied to discern evidence for shared and interpretable liability between ASD and co-occurring traits. These findings expand upon the current understanding of the complex genetics regulating ASD and reveal insights of neuronal brain disruptions potentially driving development and manifestation.

**Supplementary Information:**

The online version contains supplementary material available at 10.1186/s12888-024-06392-w.

## Highlights

Multivariate GWAS resulted in 637 significant ASD associations (*p* < 5e-8), among which 322 are reported for the first time.

The novel associations mapped to known SFARI ASD genes *CADPS*, *MAPT* and *NEGR1* and novel ASD genes *KANSL1*, *NSF* and *NTM*, associated with immune response, synaptic transmission, and neurite growth, potentially driving the gut brain-barrier hypothesis underpinning ASD development.

CuONPs induce co-occurrence of autophagy activation and autophagic flux blockade.

Mendelian randomization analyses found that genetic liability for ASRD and DBT have causal effects on the risk of ASD while genetic liability for ASD have causal effects on the risk of ADHD, BIP, WA, MDD and SCZ. Bidirectional genetic liability causal effects were confirmed between ASD and ADHD childhood.

**Supplementary Information:**

The online version contains supplementary material available at 10.1186/s12888-024-06392-w.

## Introduction

ASD spectrum disorders (ASD) is an umbrella term for a group of heterogeneous neurodevelopmental traits that manifest in early childhood. ASD is a complex disorder with both genetic and environmental risk factors [10, 30, 45]. The diagnosis of ASD is based on its key characteristics including difficulties in social communication and interaction, restricted and repetitive behaviors, hyperactivity and divergent responses to sensory inputs. The most common co-occurring traits in autistic persons are attention deficit hyperactivity disorder (ADHD), ADHD childhood, anxiety, bipolar (BP), depression, epilepsy, obsessive compulsive disorders (OCD) and stress related traits, all of which share overlapping diagnostic attributes and challenging symptoms with ASD [30, 57]. According to US data, autistic children tend to fare less well in educational attainment (EA) and about one in three have a reduced intellectual ability, as defined by intelligence quotient (IQ less than 70) [4, 68]. Some children with ASD having higher IQ scores also comparatively experience harder academic struggles due to co-occurring traits and difficulties in social interactions [3].

Together with recent advances in genomics technology and pivotal support from the engaged ASD community, 1,162 genes are currently implicated with ASD development and these are curated in the SFARI [2, 19, 52] gene module. These genes, with varying degrees of effect, are scored using the Evaluation of ASD Gene Link Evidence (EAGLE) framework [61]. Surprisingly, while it is known that common variants contribute to most of the genetic background [18], only a few robust genetic associations have been recently reported. Most of these are attributed to the landmark study conducted by Grove and colleagues, employing a large Danish cohort with 18,381 ASD cases and 27,969 controls, where 12 significant variant associations were reported [19].

Given that there is overlap in symptoms between ASD and ADHD, recent genetics studies found shared genetic factors underlying ASD and ADHD [40, 41, 50], with partial concordance between bidirectional colocalization single nucleotide variants (SNPs). However, these studies were limited to general ADHD (onset age 10+), and not childhood ADHD. Astoundingly many (47% median) autistic children have reported one or more gastrointestinal (GI) symptoms [5]. Recently, there have been promising results that link microbiome disruption and diversity [44] as a novel contributing factor to ASD. While Grove and colleagues found that 7 of the 12 ASD SNP associations have similar significance towards EA and psychosis traits depression and schizophrenia [19], still little is known concerning the joint liability and the shared genetic mechanisms between ASD and ASD co-occurring traits including ADHD, ADHD childhood, anxiety-stress related disorder (ASRD), bipolar, disruptive behavior disorder (DBD), EA, epilepsy, inflammatory bowel disease (IBD), major depression, obsessive compulsive disorder (OCD) and schizophrenia (SCZ). Respectively, the 11 co-occurring trait summary statistics are retrieved from large reputable cohorts, listed in Table 1 and Supplementary Table 1.

To attenuate the genetic knowledge gaps in ASD and expand the exploration of potential shared co-occurring trait genetic associations, this study performed multivariate genome-wide association study (GWAS) with summary statistics from ASD and 11 co-occurring traits from large reputable cohorts. To achieve this, colocalization (coloc) was systematically applied to test the robustness between the shared variants and traits [75]. Mendelian randomisation (MR) was further applied, using the multivariate variants and the essential traits, to assess liability relationships between ASD and the selected co-occurring traits [6, 55]. This study seeks to further clarify functional, regulatory and tissue type differentiation with enrichment and integration of quantified trait loci (QTL) while validating our key findings with independently sequenced genomes from the GEMMA cohort [70].

## Methods and materials

**Fig. 1:**
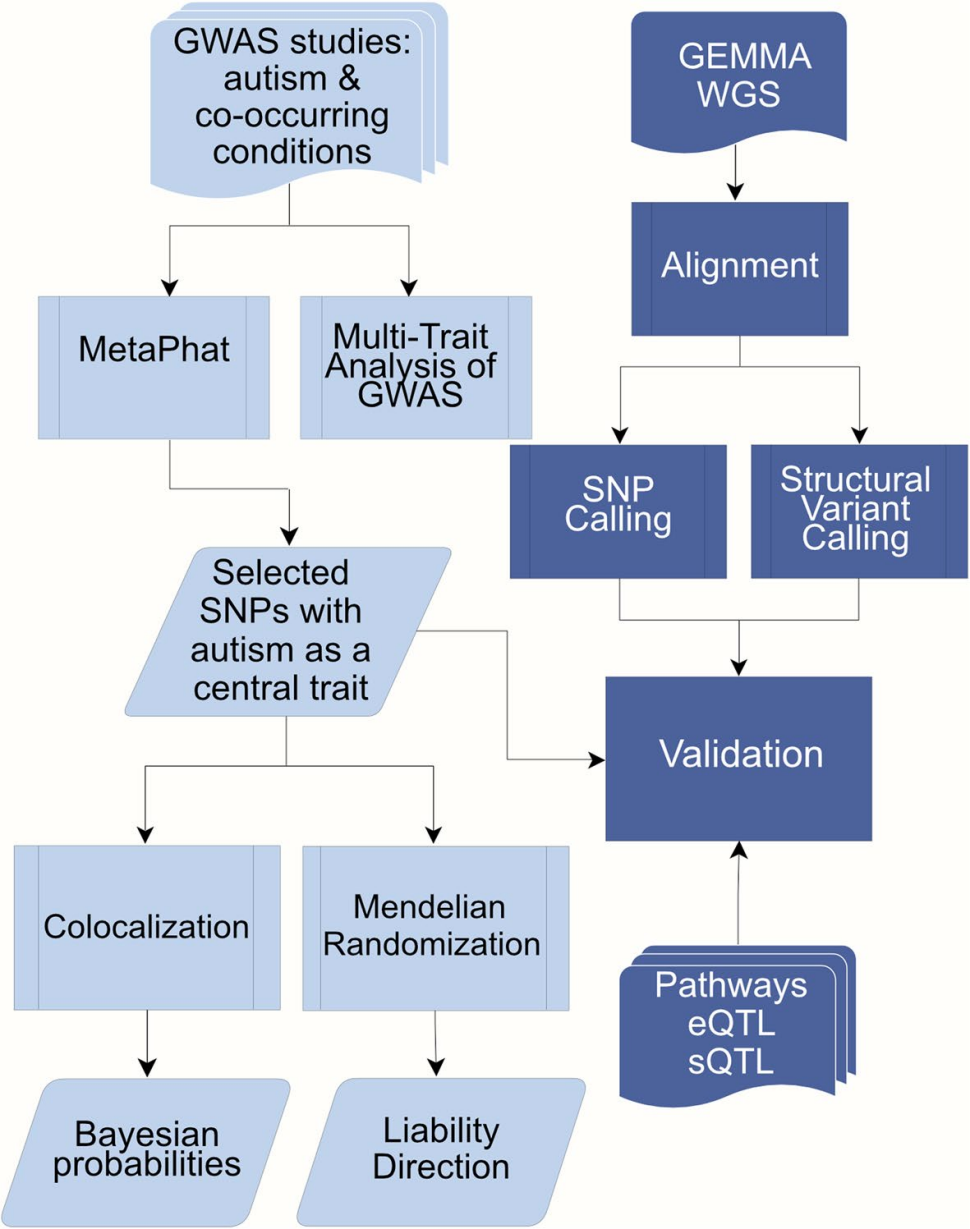
Workflow for the analyses conducted in the study. Multivariate GWAS was performed on selected GWAS studies including ASD and 8 co-occurring traits: ADHD, ADHD childhood, bipolar, anxiety, disruptive behaviour, educational attainment, major depression and schizophrenia. 37 SNPs were selected and evaluated with Colocalization and Mendelian Randomization. Further validation of these SNPs utilized pathway and EBI eQTL/sQTL catalogs as well as the GEMMA -study. The GEMMA whole genome sequencing (WGS) processing included variant calling to infer structural and single nucleotide variants (SVs and SNVs) present in the samples

GWAS summary statistics for ASD and ADHD were collected from the Psychiatric Genomics Consortium (PGC) and iPSYCH [49, 65] studies. Education attainment [47] summary file was collected from the Social Science Genetic Association Consortium (SSGAC). Additional ASD co-occurring traits, selected based on LDSC (LD Score Regression) genetic correlation (*p*-value < 0.05) with ASD, include ADHD childhood, bipolar (BP), anxiety-stress disorder (ASRD), disruptive behaviour (DBD), major depression (MDD) and schizophrenia (SCZ), with sample sizes ranging from 31,890 − 765,283 are shown in Table 1 (additional details including doi references listed Supplementary Table 1). To estimate potential sample overlaps, pairwise LDSC intercepts with ASD are calculated and reported in Supplementary Table 1. Summary statistics are joined, yielding 4,525,476 SNPs, and applied in a multivariate GWAS setting. Follow-up analysis includes decomposition aiming to detect the most important traits while colocalization and Mendelian randomisation analysis are conducted to explore shared liability as shown in Fig. 1.

### Multivariate GWAS and determination of central traits

Multivariate GWAS on ASD and ASD co-occurring traits were performed using metaPhat/metaCCA software that performs multivariate analysis by implementing Canonical Correlation Analysis (CCA) for a set of univariate GWAS summary statistics [12, 36, 58]. The objective of metaCCA is to find the optimal genetic effect combination that is maximally correlated with a linear combination of the trait variables. ASD multivariate central traits are identified by MetaPhat decomposition based on iterative tracing of *p*-values (p) from trait subsets (relative to 5e-8) and Bayesian Information Criterion (BIC) [62] representing model fit. Essentially, driver trait(s) are the subsets of the multivariate association that drives the p-value, and without the drivers, the multivariate association is no longer significant (*p* > 5e-8). Similarly, as the decomposition processing is exhaustive (iterates from k to 1), an optimal subset is identified by comparing BIC values [36]. For simplicity, the central traits are the union of the driver and optimal BIC traits. Multi-Trait Analysis of GWAS (MTAG) [71], a high performance multivariate-GWAS that addresses sample overlap, is additionally performed for validation.

### Genetic annotations, pathway enrichment and validation

SFARI Base Gene resource, GeneCards and GWAS catalog were used to assess the novelty of variants and genes associated with ASD [2, 39, 60]. snpXplorer was applied towards SNP annotation [69]. Reactome and WikiPathway databases pathway enrichments were evaluated with the Enrichr tool [31]. Human organ and cell type systems enrichment analysis, encompassing 1,466 tissue-cell type and single-cell RNAseq panels, was conducted using WebCSEA [13, 33]. eQTL and sQTL were assessed within the QTL catalog, via FIVEx portal [32].

### Colocalization analyses

Colocalization was performed for the selected multivariate ASD SNPs to assess if the associated variants in the locus are shared genetically between ASD and the 8 co-occurring related ASD traits to account for erroneous results that may follow from analyzing individual SNPs. Errors can occur when a SNP associated with trait 1 and trait 2 are in linkage disequilibrium (LD). The analyses were performed using the R package coloc [20, 33].

The colocalization analysis was conducted using the absolute base factor colocalization method (coloc.abf), which is a Bayesian colocalization analysis method. A region size window of 100KB (± 50 KB flanking the SNP position) was selected to comprehensively span potential LD and regulatory elements [53]. The different hypotheses tested include: H0 (no liable variant), H1 (liable variant only for trait 1), H2 (liable variant only for trait 2), H3 (two separate liable variants), H4 (common liable variant shared between the traits). As recommended [74], default setting prior probability thresholds were applied: 1e-4 for H1, H2 and H3 and 1e-5 for H4 while posterior probability (H4 > 90%) is conservatively applied to estimate shared liability.

### Mendelian randomization analyses

Mendelian Randomization analyses (MR) was conducted on the selected multivariate GWAS SNPs based on their assigned central traits, to explore the liability, direction and independent (reverse causation) relationships between ASD and its related traits [51]. Instrumental strengths, approximated with F1 score > 10, were calculated using SNP effect and standard error values [6, &nbsp;49]. To account for the potential biases due to participant overlap between cohorts, the lower bound (95% confidence interval) of the F1 was calculated [9]. The analyses were performed using the platform TwoSampleMR [6].

### Whole genome sequencing

The results were validated using yet unpublished data from the EU Horizon2020 GEMMA research project with genotype variant calls in 112 (49% female) WGS samples with 45 ASD probands (42% female) from the GEMMA prospective cohort [70]. These samples, assayed on whole blood and collected during enrollment, were sequenced with 30-40X coverage on Illumina NovaSeq 6000 platform. Data was aligned to GRCh38 reference genome using bwa mem v0.7.17 [34] and reads were sorted and duplicates marked with samtools v1.12 [35]. Quality control was performed with omnomicsQ -software [20]. For variant calling DeepVariant v1.4.0 [54] was utilized and variants were annotated with Variant Effect Predictor [43] version 112.0.

### Statistical analysis

All statistical analyses were performed using R 4.2.2 software and available as R markdown results in the github project (https://github.com/jakelin212/mvasd_gwas*).* Genome-wide association is called on the standard and strict p-value threshold of 5e-8 (-log10 7.3), to account for multiple testing based on the assumption of about 1-million independent tests [56]. To assess SNP allele proportional differences for validation, the phi coefficient is computed, and statistical significance was determined using Chi-square test. Fisher’s exact test was used when Chi-square assumptions were not met. Bonferroni correction is assessed to account for multiple testing of the multivariate GWAS involving 9 traits (*p* < 5.5e-9; -log10(p) > 8.25).

## Results

### GWAS summary statistics

GWAS summary statistics for ASD and ADHD were collected from the PGC and iPSYCH [49, 65] studies. Education attainment [47] summary file was collected from the Social Science Genetic Association Consortium (SSGAC). Altogether, using summary statistics, 11 ASD co-occurring traits were assessed for genetic correlation with the landmark ASD study [19], the largest genetic correlation values, as computed by LDSC [8], were between ASD and ADHD (rg = 0.535), followed by MDD (rg = 0.505) and ADHD childhood (rg = 0.478). Shown in Table 1 below, 8 traits are shown to be genetically correlated with ASD (*p* < 0.05) and additional details of all traits are shown in Supplementary Table 1.

**Table 1:** Data of ASD and 8 genetically correlated traits (*P* < 0.05, calculated from LDSC), reported SNP heritability (H2), genetic covariance and covariance scores (standard errors) are presented and applied towards multivariate-GWAS to explore multivariate associations and additional trait refinement. More details and excluded traits are listed in supplementary table 1

Trait	Heritability (H2)	Genetic correlation (rg)	*P*	Genetic covariance	Intercept
ASD^a^	0.118 (0.010)	na	na	na	na
ADHD^b^	0.140 (0.010)	0.535 (0.041)	1.44e-38	0.074(0.006)	0.233 (0.009)
ADHDCHILD^c^	0.235 (0.015)	0.478 (0.052)	5.21e-20	0.104 (0.012)	0.260 (0.007)
ASRD^d^	0.280 (0.027)	0.441 (0.079)	2.22e-08	0.090 (0.015)	0.221 (0.006)
Bipolar^e^	0.068 (0.003)	0.219 (0.041)	9.67e-08	0.026 (0.005)	0.032 (0.006)
DBD^f^	0.100 (0.012)	0.186 (0.07)	0.008	0.026 (0.010)	0.179 (0.006)
EA^g^	0.321 (0.009)	0.207 (0.025)	9.95e-17	0.053 (0.007)	−0.005 (0.007)
MDD^h^	0.090 (0.004)	0.505 (0.003)	2.78e-36	0.037 (0.003)	0.155 (0.005)
SCZ^i^	0.240 (0.007)	0.258 (0.035)	7.87e-14	0.070 (0.010)	0.018 (0.007)

*Abbreviations*: *ADHD *Attention Deficit Hyper Disorder, *ADHDCHILD *ADHD childhood, *ASRD *Anxiety-Stress Disorder,*DBD *Disruptive Behaviour Disorder, *EA *Education attainment, *MDD *Major Depressive Disorder, *SCZ * Schizophrenia

^a^Grove et al. [19] (PMID: 30804558)

^b^iPSYCH + deCODE + PGC, Demontis et al. 2023 (PMID: 36702997)

^c^iPSYCH, Rajagopal et al. 2022 (PMID: 35927488)

^d^iPSYCH excluding ASD cases Meier et al. 2019 (PMID: PMC6537792*)*

^e^Discovery excluding UKB Mullins et al. 2021 (PMID: 34002096)

^f^Demontis et al. 2021 (PMID: 33495439)

^g^Discovery cohorts excluding 23andme, Okbay et al. [47] (PMID: 35361970)

^h^PGC excluding UKB, Wray et al. 2018 (PMID: 29700475)

^i^PGC Wave 3, Trutbetskoy et al. 2022 (PMID: 35396580)

### Multivariate ASD central trait SNPs, pathway and organ tissue enrichment

Multivariate GWAS was performed with ASD together with its genetically correlated traits, ADHD, ADHD childhood, ASRD, bipolar, DBD, EA, MDD, and SCZ (Table 1) and 637 (*p* < 5e-08) SNP associations were found, including 322 variants that are reported for the first time for any trait (Supplementary Table 6) according to GWAS catalog. Two associations (rs2388334 and rs1452075) intersected with the twelve associations identified in the landmark common genetic variants of ASD study [19]. When assessed at the gene level, all 12 were concordant (as indicated in STable 6). Decomposition implemented in MetaPhat, using stepwise tracing of *p*-value and Bayesian information criteria (BIC) contributions [36, 62], identified 37 ASD central trait SNPs where 16 were identified with multivariate GWAS approach (all SNPs *p* < 5.5e-09; min (-log10(p) 8.67), listed in Supplementary Table 2). These 37 multivariate ASD SNPs, 17 of which had previously been reported in existing GWAS studies, mapped to 35 genes (Table 2) and confirmed that 8/35 (*ARHGAP32*, *CADPS*, *CUL3*, *KANSL1*, *MACROD2*, *MAPT*, *MSRA* and *NEGR1*) are known curated SFARI genes, with ASD susceptibility EAGLE scores < = 3 (indicating limited evidence) [61]. The variant rs538628 within the *NSF* gene, a regulator of AMPA receptor endocytosis and critical for mediating glutamatergic synaptic transmission [25], along with the variant rs62061734 in the *MAPT* gene, are identified to associate with the optimal central traits of ASD, EA and SCZ (*MAPT* variant rs62061734 *p* = 3.98e-31, *NSF* variant rs538628 *p* = 1.99e-27, Supplementary Table 2, trace plots are provided in supplementary data). Notably, *NSF* was previously implicated only in mouse models exhibiting ASD-like behaviors [76]. Shown in the same table, MTAG [71] multivariate GWAS validation was performed to address iPSYCH cohort sample overlaps between ADHD and ASD [40, 41] subjects where similar results were found (*MAPT* variant rs62061734 *p* = 1.99e-20, *NSF* variant rs538628 *p* = 5.37e-18).

**Fig. 2:**
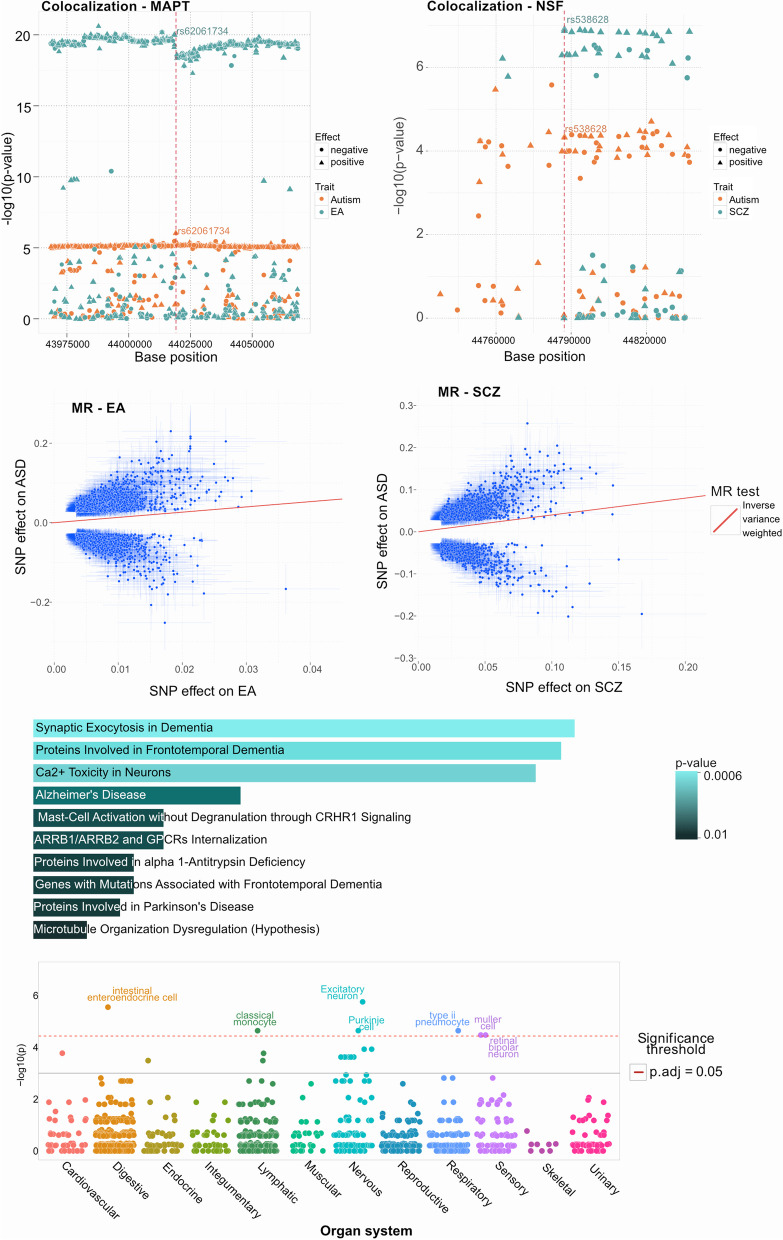
Results from the post GWAS analysis of the 37 selected SNPs. **a,b**) Colocalization processing using the original summary statistics of ASD and EA for (a) rs62061734 (*MAPT*, failed colocalization with H4 probability 8.19%, p = 0.09), ASD and *NSF* for (b) rs538628 (*NSF*, SCZ passed colocalization with H4 probability 94%, p = 1.1e-05), depicting supporting regional SNPs (x-axis) and their negative log10 *p*-value (y-axis) and effect direction (circles negative, triangles positive). **c,d**) Mendelian randomization (MR) results using inverse variance weighted (IVW) -method for association of ASD SNP effects (y-axis) and c) EA and d) SCZ effects (x-axis). **e**) Pathway analysis for the genes associated with the selected SNPs shows enrichment in processes related to neurons using Reactome database. The length of the bar represents the significance of that specific gene-set or pathway and the color indicates the significance of the pathway. Details of the pathways and genes with their associated p-values are listed in Supplementary Table 8. **f**) Organ system enrichment was applied using WebCSEA, using the selected 37 multivariate gene associations and found enrichment (*p* < 1e-03) with the ASD relevant digestive, nervous and sensory organ systems as well as lymphatic and respiratory systems

Shown in Supplementary Table 7, Fig. 2e and Supplementary Fig. 3, pathway enrichment using the 35 associated genes was performed with Enrichr [31]. Nervous systems development (GO:0007399) was found to be the most significant (*p* = 1.73e-08) while neural and microtubule structural related pathway hits from Reactome [16] and WikiPathways [46] featured pathways were Inclusion Body Myositis (*MAPT* and *PSEN1*, *p* = 1.27e-04) and COPII-mediated Vesicle Transport (*NSF* and *SERPINA1*, *p* = 4.69e-03). Enrichment analysis was conducted using the WebCSEA tool, which identified statistically significant associations (Fig. 2f, *p* < 1e-03) with the following human organ systems: digestive, nervous, sensory, lymphatic, and respiratory. As shown in Supplementary Fig. 4, the most enriched tissue types are related to cerebrum, cortex, intestine and blood related components discerned from 1,355 tissue-type (TS) as well as data from the human brain single cell project [33].

**Table 2:** Multivariate GWAS ASD-central SNPs tested with coloc and MR tests towards the identified ASD central traits, with all 8 traits passing MR and 19 gene regions/traits pairings passed Coloc (H4 > 90%), indicated with +. Coloc and MR details are additionally listed in supplementary tables 3 and 4. The order of the central traits are determined by *p*-value importance during decomposition processing. Known GWAS associations (17/37) are marked as * while SFARI ASD gene members (8) are in bold

Rsid	Gene	Chr: pos: ref > alt	SNP consequence	Central traits
rs6699841	**NEGR1**	1:72645850:A > G	intergenic	EA, ASD
rs67980110	ENSG00000237435	1:96470851:T > C	regulatory	EA, ASD, ADHD
rs2391769*	NA	1:96978961:A > G	intergenic	ASD, ADHD
rs58378462+	ENSG00000221849	2:104138639:A > G	intron	ASD, EA+, ADHDCHILD, ADHD
rs11897599	MRPS18BP2	2:140449566:A > G	downstream	EA, ASD, ADHD
rs78826721+	NDUFS1	2:207002314:A > G	intron	SCZ+, EA, ASD
rs6748341*	**CUL3**	2:225377574:T > C	regulatory	ASD, EA, SCZ
rs1452075*	**CADPS**	3:62481063:T > C	intron	EA, ASD
rs62243489+	**CADPS**	3:62482927:T > G	intron	EA+, ASD
rs6806355+	NA	3:70488292:T > G	intergenic	BP, SCZ+, EA+, ASD
rs35544582+	SLC30A9	4:42044036:A > C	intron	EA+, ASD
rs67779882	NA	5:92488009:A > G	intron, non_coding	EA, ASD, ADHD
rs406413*	ENSG00000246316	5:113898581:T > C	intron	EA, ASD
rs2388334*	ENSG00000271860	6:98591622:A > G	intron	BP, EA, ASD
rs6999466+*	**MSRA**	8:10265712:A > G	intron	MDD, EA+, ASD
rs877116*	ENSG00000253695	8:10712945:T > G	intron	ASD, ASRD
rs2409743*	ENSG00000270076	8:11070360:T > G	intron, non_coding	ASD, ASRD
rs2409784+	BLK	8:11396856:A > C	intergenic	ASD, ASRD+
rs11775333	NA	8:142637867:T > C	regulatory	EA, ASD
rs11143599	ENSG00000221844	9:76101777:T > G	intron, non_coding	BP, ASD
rs1848797+*	ENSG00000238280	10:64552934:A > G	intron	BP+, EA, ASD
rs12761761*	BNIP3	10:133775375:T > C	downstream	EA, ASD
rs2237943	SERGEF	11:17838248:T > C	regulatory	EA, ASD, ADHD
rs4609618	**ARHGAP32**	11:128818792:A > C	intergenic	ASD, ADHD
rs568828+	NTM	11:131732259:T > G	intergenic	ASD, SCZ+, ADHD+
rs177413+	PSEN1	14:73683194:T > C	intergenic	BP+, ASD, EA, SCZ
rs736281*	NA	14:94287830:T > C	intron	EA, ASD
rs28929474*	SERPINA1	14:94844947:T > C	intron	EA, ASD
rs62065453+*	ENSG00000131484	17:43573419:A > G	regulatory	ASD, EA+, SCZ+
rs62057107+*	CRHR1	17:43896032:T > C	intergenic	ASD, EA+, SCZ+
rs62061734+*	**MAPT**	17:44018488:T > C	intron	ASD, EA+, SCZ
rs2696633+	**KANSL1**	17:44270059:T > G	intron	ASD, EA+, SCZ
rs538628*	NSF	17:44787313:T > C	regulatory	ASD, EA, SCZ+
rs1792709*	ENSG00000206129	18:53768975:A > G	intron	ASD, SCZ
rs6079546	**MACROD2**	20:14716738:T > G	intergenic	MDD, EA, ASD
rs6035835	XRN2	20:21271669:A > G	intergenic	ASD, ADHDCHILD, ADHD
rs9974470	ENSG00000249209	21:35012066:A > G	intron	EA, ASD

## Colocalization analyses

Colocalization analysis was conducted on the 37 multivariate SNP associations identified to contain ASD as a central trait. The comparative analysis was performed on the relevant mapped gene window, from start to end while adding 25 KBs on both ends to cover regulating and promoter regional elements. For the two SNPs that did not map to a gene, the window size used for the colocalization analysis was 100 KB (± 50 KB), estimated and derived from the gene median length of 24KB [17]. Additional information concerning the number of regional LD adjusted SNPs applied to the colocalization test is shown in Supplementary Table 3.

A total of 19/37 SNPs showed strong evidence for a common liability variant with ASD (H4 > 90%, details shown in Supplementary Table 3) and the traits having common ASD liable variants included EA (9), SCZ (6), BP (2), ADHD (1) and ASRD (1). Notably, SNP rs62061734, mapping to the *MAPT* gene and rs538628, mapping to the *NSF* gene had H4 of 99% for EA and SCZ, respectively (shown in Fig. 2a-b) while SNP rs568828, mapping to the *NTM* gene had H4 of 99% for SCZ and ADHD (Supplementary Table 2).

### Mendelian randomization analyses

Mendelian randomization analysis was conducted for the 8 traits genetically correlated (Table 1) with ASD. The lead SNPs, with F1 scores > 25 (listed in Supplementary Table 4, where > 10 is considered strong [48] were found to lend significantly increase probability of ASD (*p* < 0.001 both Inverse Variance Weighted (IVW)-method and MR-Egger (EA and SCZ are shown in Fig. 2c-d), accounting for horizontal pleiotropy and multiple testing with Bonferroni correction of 8 traits). Based on TwoSampleMR Steiger [22] test for directionality and shown in supplementary Table 4 A, genetic liability to ADHD childhood (*p* < 2.44e-116), ASDR (*p* < 9.08e-166) and DBD (*p* < 1.20e-45) were found to have causal effects on the risk of ASD. Shown in Supplementary Table 4B, genetic liability to ASD (*p* < 4.1e-115) were found to have causal effects on the risk of ADHD, ADHD childhood, BIP, EA, MDD and SCZ. The related MR results adhere to the MR-STROBE guidelines [64].

### Validation

To assess the impact of the reported multivariate associations on expression (eQTL) and splicing regulatory quantitative trait loci across tissues, the majority (22/37 eQTL, 24/37 sQTL, details listed in Supplementary Table 9) of the associations found are cited in the EBI QTL Catalog [28] where they associate (adjusted *p* < 0.05) with adipose, brain and neuron tissues. Furthermore, filtering on GeneCards [60] curations, the presented ASD central genes are enriched with systems related to gut, microbiome, intestinal immune, enteric nervous and central nervous systems (Supplementary Table 5).

Additionally, the distribution of these ASD-central trait related SNPs in 112 (49% females; 45 ASD probands (42% females) prospective from-birth GEMMA [70] cohort participants was investigated. SNP distribution differences were for variant rs568828, mapped to *NTM* and rs62243489, mapped to *CADPS*. The *NTM* gene, previously associated with emotional learning deficits in murine models [42], encodes neurotrimin, while *CADPS* encodes a neural/endocrine-specific membrane protein regulating calcium. The *NTM* SNP (rs568828) was present in 42 of 45 probands (92%) compared to 100% of controls (67 of 67). In contrast, *CADPS* SNP (rs62243489) was found in 19/67 controls (28%) and enriched in 21/45 probands (47%). As listed in Supplementary Table 8, the phi coefficient for *NTM* between probands and controls was 0.2 (*p* = 0.062), while for *CADPS*, it was − 0.19 (*p* = 0.047). When stratified by sex, the phi coefficient for *NTM* in males was 0.15 (*p* = 0.456) and 0.27 (*p* = 0.040) in females. For *CADPS*, the phi coefficients were − 0.18 (*p* = 0.182) in males and − 0.20 (*p* = 0.140) in females. Notably, the *NEGR1* gene (variant rs6699841), involved in neuron growth regulation, showed a phi coefficient of −0.27 (*p* = 0.040) in males (24/26 cases; 22/31 controls), while in females, the coefficient was 0.26 (*p* = 0.084; 12/19 cases; 31/36 controls). For the variant of *NEGR1*, the opposing phi directions between sexes resulted in a phi coefficient of −0.01 (*p* = 0.908) in the full dataset. In addition, logistic regression was performed for the specific variants of *NTM*, *CADPS* and *NEGR1*. The results were not significant for the full cohort (adjusted for sex) or in models stratified by sex.

## Discussion

Using multivariate statistical learning approaches, this study constitutes the largest and most comprehensive genetically correlated multi-trait GWAS analysis with summary statistics performed on ASD and its genetically correlated traits; ADHD, ADHD childhood, ASRD, bipolar, DBD, EA, MDD, and SCZ to explore the underpinnings driving the complexities in ASD. 37 associations containing ASD as a central trait were discovered, with 16 of these associations were detected only due to the increased statistical power of this multivariate GWAS analysis (lowest univariate summary statistics p-value from all traits > 5e-08, and 12/16 confirmed with the MTAG tool [71], Supplementary Table 2). Interestingly, a previous study using electronic health records of covering nearly 5,000 ASD cases found three subclusters of comorbidity trajectories, first characterized by seizures, then auditory disorders/infections and the third cluster by psychiatric disorders. Due to the complexity of ASD development, a fourth group was described as could not be further resolved. The presented subclusters potentially align well with our ASD central trait sets pertaining to SCZ signals with seizures and psychiatric disorders such as ADHD and intellectual development underpinning EA [15]. Enrichment analysis confirmed that the multivariate ASD association results are related to neuron and gut tissues and developmental pathways as well as inflammation and microbiome domains, further underscoring the intersection of genome and microbiome as well as supportive of the gut-brain axis hypothesis associated to ASD [11, 44]. Surprisingly, genetic correlation performed on LDSC indicated that ASD and IBD are not related (Supplementary Table S1, rg = −0.059; *p* = 0.44), a recent report highlighted potential evidence for comorbidity between parental, particularly maternal preexisting IBD onsets and their children developing ASD [59]. Using the multivariate ASD central trait gene sets, based on comprehensive human tissue cell type and single cell data [13, 33] analysis, enrichments were detected with digestive, nervous, and sensory organ systems (Fig. 2f). At the tissue cell type level and further supporting the gut-brain axis and blood brain barrier, the analysis detected enriched ASD relevant signals related to brain, adipose and gut eQTL/sQTL (Supplementary Table 9) tissue panels.

Overall, the identified ASD traits passed MR with strong F1 measures and significantly contributed to improve the future construction of meta psychiatric based ASD polygenic scores [27], shown to improve prediction relative to standard PRS in other complex traits such as coronary heart disease and type 2 diabetes [37, 67]. The MR results were consistent after calculation of lower bound F1 (all scores > 25, Supplementary Table 4), computed to consider potential biases from cohort sample overlaps [9]. MR Steiger tests for directionality revealed that genetic liability to ASDR and DBD were found to have causal effects on the risk of ASD. These multivariate ASD associations mapped to genes, including *MAPT* and *NSF* which are known to involved in biological pathways linked to neural disorders such as infantile epilepsy [66] and Parkinson’s Disease [7, 14]. Interestingly, colocalization tests for the *MAPT* region indicated shared genetic risk between only EA and ASD (H4 99%), while that for the *NSF* gene did not associate with EA, instead associated with SCZ (H4 94%), suggesting intra region heterogeneity that demands future investigation. With respect to ASD, the *KANSL1*, *BNIP3*, *CADPS* and *NEGR1* genes have been implicated with immune and microbiome features [11] and behavioral developments [63]. Similarly, a recent study from Arenella and colleagues reported genetic factors between ASD and various immune phenotypes including *KANSL1* associating with lymphocyte counts as well as *MAPT* associating with eosinophil counts, further supporting the role of the inflammation pathway in ASD development [1].

The most common traits in our set of 37 associations that passed colocalization with ASD were EA (9), SCZ (6) and BP (2). It is known that the diagnosis for ASD and ADHD, particularly ADHD manifestation in young children, is similar with symptomatic issues concerning hyperactivity and attention span [29]. While a previous study has performed comparison of genetic and functional enrichment of associations between ASD and ADHD [50] GWAS resources, this study further complements their results by inclusion of other ASD co-occurring traits, including ADHD and ADHD childhood as well as EA. Interestingly, ASD and ADHD have both been linked with dysbiosis disruption in microbiome composition and function, gastrointestinal and bowel habits issues [44].

As part of validation, clustering and distribution proportion differences based on the ASD identified SNP associations were detected between probands and non-autistic subjects on genomes from the GEMMA cohort [70]. Our validation results were performed on the (112, 45 ASD probands) samples currently available in GEMMA. Notably, *NEGR1* (rs1432639), a neuronal growth regulator known to associate with migraine, depression and seizures [24, 26, 73], the significant phi coefficients were negative for males and positive for females. Interestingly, a previous study pertaining to prenatal stress found increased *NEGR1* expression in the hippocampus of female rats but not in males [72]. To improve on the specificity and clinical value of the identified traits, a follow-up application of MR with specific expression/protein quantitative loci (tissue/cell type e/pQTL as applied in T1D drug candidate discovery [21] with genes such as *CADPS*, *NTM* and *NEGR1* could further reveal molecular and translational insights towards ASD heterogeneity including the high vulnerability subgroup characterized by seizures [15]. While the validation statistical power was limited by the relatively small sample size, nevertheless the independent and deep sequencing data has allowed the harvesting of interesting observations concerning the distribution of ASD-central trait associations in probands as compared to controls. In addition, the GEMMA validation results should be taken with caution as the population structures (PCs) were not included as covariates due to availability. The upcoming release of additional omics data from GEMMA and other studies, including longitudinal microbiome, metabolome, and methylation datasets, will significantly increase statistical power and enable more detailed temporal analyses. The data will help confirm molecular changes along the gut-brain axis, shedding light on the genetic patterns that contribute to the heterogeneity, development, and comorbidities of ASD. Another limitation of our multi-trait GWAS is that the selection of ASD co-occurring traits is not exhaustive; given the complexity of ASD development, there may be other genetically correlated traits that have not yet been tested at the appropriate population level, warranting consideration and inclusion in future studies. The MR Steiger results on causality need to be taken with caution as unmeasured confounding effects may distort the exposure genetic liability relative to the outcome [38].

## Conclusion

Our study represents the largest multivariate GWAS on ASD to date, combining ASD with eight genetically correlated trait GWAS summaries. We performed systematic decomposition to identify novel genetic associations related to ASD and ASD co-occurring traits. Mendelian randomization testing revealed that genetic liability for ADHD childhood, ASRD and DBD has causal effects on the risk of ASD. Colocalization analysis further confirmed shared genetic risks with ASD, showing enrichment patterns in brain tissues and cell types associated with neurodevelopment, and lending additional support to the gut-brain axis hypothesis.

## Supplementary Information

Supplementary Material 1.

Supplementary Material 2.

Supplementary Figure 1. Genetic correlation of the traits included in the analysis. ASD = Autism Spectrum Disorder; ADHD = Attention Deficit Hyper Disorder; ASRD = Anxiety-Stress Disorder; DBD = Disruptive Behaviour Disorder; EA = Education attainment; MDD = Major Depression Disorder; SCZ = Schizophrenia. Supplementary Figure 2*. P*-value and BIC decomposition processing of MAPT and NSF to identify ASD central traits. ASD=Autism Spectrum Disorder; ADHD=Attention Deficit Hyper Disorder; ASRD=Anxiety-Stress Disorder; DBD=Disruptive Behaviour Disorder; EA=Education attainment; MDD=Major Depression Disorder; SCZ=Schizophrenia. Supplementary Figure 3. Pathway analysis using the WikiPathway database also highlights neuronal processes, with bar length and color indicating significance. More details listed in Supplementary Table 8. Supplementary Figure 4. Tissue and cell (TS) type enrichment using WebCSEA and the list of the 22 central trait genes found that the most enriched tissues are related to cerebrum, cortex and small intestine related tissue types. Lake 2017 refers to data from human brain single cell analysis project (https://pubmed.ncbi.nlm.nih.gov/29227469/) while HCA stands for histologic chorioamnionitis, an intrauterine inflammatory trait. Supplementary Figure 5. ASD multivariate GWAS associations within the MAPT H1/H2 haplotype, 17q21 arm region, are presented in a Manhattan plot, in the context of Grove et al. GWAS results. Significance thresholds for *p*-values of 1e-05 indicated in blue and 1e-08 in red. Significant SNPs highlighted in green show rs62061734 (MAPT), rs269633 (KANSL1) and rs538628 (NSF).

Supplementary Table 1. Data and sample details of ASD and 8 genetically correlated traits (*P* < 0.05, calculated from LD Score Regression (LDSC)) are presented and applied towards multivariate-GWAS. Data from four excluded traits are additionally shown. Supplementary Table 2. 37 multivariate associations are identified with ASD as a central trait where 17/37, shown with asterisk are previously reported in the GWAS Catalog and in bold, 8 genes are identified as SFARI ASD genes. Supplementary Table 3. 19 gene regions/trait pairings passed coloc (Posterior Prob H4 > 90%, Shown in bold) called on coloc.abf with a window size of ± 50 KB flanking the SNP locus. Supplementary Table 4. (A) Mendelian randomization (MR) results for ASD as outcome and related traits. (B) MR where ASD is the exposure and related traits are the outcome. Supplementary Table 5. MV associated genes are found in systems curated/implicated with gut microbiome and neural systems from GeneCards. Supplementary Table 6. List of 637 Significant SNPs (*p* < 5e-8), with 315 already reported in the GWAS catalog, identified by MetaPhat multivariate-GWAS using ASD and 8 genetically correlated trait summary statistics. Supplementary Table 7. A) 108 enriched (*p* < 0.05) Go terms are annotated and (B) 46 pathways on WikiPathway C) KEGG D) Reactome resources e) Tissue from the list of multivariate ASD SNPs found enrichments in neuron and nervous systems related data. Supplementary Table 8. ASD central SNP alleles are mapped to GEMMA genotypes called from 112 (49% females) WGS samples (45 (42% females) ASD probands). Phi coefficients are calculated between allele proportions where Chi-square test is applied to assess statistical importance. Indicated with *. Fisher's exact test is applied when Chi-square assumptions are violated. Supplementary Table 9. eQTL and sQTL related results of the ASD central associations relative to brain and nervous systems from EBI QTL catalog are captured via https://fivex.sph.umich.edu/. Study URLs are listed at the bottom of the table.

## Data Availability

All data generated or analyzed during the study are included and additionally available upon request. For scripts, please see: https://github.com/jakelin212/mvasd_gwas
