# Supplementary material for: Exploring autism spectrum disorder and co-occurring trait associations to elucidate multivariate genetic mechanisms and insights
Source: BMC Psychiatry. 2024 Dec 18;24:934. doi: 10.1186/s12888-024-06392-w (PMC11658126; doi:10.1186/s12888-024-06392-w)
Supplement: Supplementary file 1 — Supplementary Material 1. [file 12888_2024_6392_MOESM1_ESM.docx]

**STROBE-MR checklist of recommended items to address in reports of Mendelian randomization studies**^1^ ^2^

| **Item No.** | **Section** | **Checklist item** | **Page No.** | **Relevant text from manuscript** |
| --- | --- | --- | --- | --- |
| 1 | **TITLE and ABSTRACT** | Indicate Mendelian randomization (MR) as the study’s design in the title and/or the abstract if that is a main purpose of the study | 1 | MR is not indicated in the title as multi-variate genetic association analysis is the main purpose of the study. MR is an important validation for related co-occurring autism conditions/traits  Mendelian Randomization analyses (MR) was conducted on the selected autism multi-trait SNPs based on their assigned central traits, to explore the liability, direction and independent (reverse causation) relationships between autism and its related biomarkers. |
|  | **INTRODUCTION** |  |  |  |
| 2 | **Background** | Explain the scientific background and rationale for the reported study. What is the exposure? Is a potential causal relationship between exposure and outcome plausible? Justify why MR is a helpful method to address the study question | 1 | Autism has been linked to multiple co-occurring conditions, such as ADHD, learning difficulties and mental health issues. The concomitant development of autism and other neurological conditions is assumed to result from a complex interplay of genetics and the environment. However, the multivariate genetic associations of autism and its co-occurring conditions are still largely unexplored. We have performed MR analyses on selected autism multi-trait SNPs to further explore the liability, direction and independent (reverse causation) relationships between autism and its related biomarkers. |
| 3 | **Objectives** | State specific objectives clearly, including pre-specified causal hypotheses (if any). State that MR is a method that, under specific assumptions, intends to estimate causal effects |  | We performed MR to further explore the shared liability of autism and co-occurring traits under instrumental variable assumptions. |
|  | **METHODS** |  |  |  |
| 4 | **Study design and data sources** | Present key elements of the study design early in the article. Consider including a table listing sources of data for all phases of the study. For each data source contributing to the analysis, describe the following: |  | Sources of the summary statistics used, including sample sizes, are listed in S. Table 1. |
|  | a) | Setting: Describe the study design and the underlying population, if possible. Describe the setting, locations, and relevant dates, including periods of recruitment, exposure, follow-up, and data collection, when available. |  | The study used publicly available summary statistics from previous studies, which included mainly individuals of European ancestry. |
|  | b) | Participants: Give the eligibility criteria, and the sources and methods of selection of participants. Report the sample size, and whether any power or sample size calculations were carried out prior to the main analysis |  | Sample sizes of the summary statistics range from 31,890-765,283. Mendelian randomization analyses were conducted on the selected autism multi-trait SNP central traits. A total of 8 autism co-occurring traits were considered. SNPs were selected based on the multivariate GWAS results having one or more co-occurring traits along with autism identified as a central trait (p < 5e-8). |
|  | c) | Describe measurement, quality control and selection of genetic variants |  | Genetic variants were selected from the summary GWAS statistics of the exposure with a p-value threshold of 0.05 and further pruned using linkage disequilibrium threshold of 0.2. Variants were then further harmonized with the genetic variants of the outcome summary statistics |
|  | d) | For each exposure, outcome, and other relevant variables, describe methods of assessment and diagnostic criteria for diseases |  | Pre-existing GWAS summary statistics from leading studies were used. Assessment methods and diagnostic criteria were previously defined as a part of the original studies. |
|  | e) | Provide details of ethics committee approval and participant informed consent, if relevant |  | - |
| 5 | **Assumptions** | Explicitly state the three core IV assumptions for the main analysis (relevance, independence and exclusion restriction) as well assumptions for any additional or sensitivity analysis |  | The MR analyses were performed under the 3 instrumental variable assumptions. 1. Instruments are associated with the exposure 2. Instruments have no confounders that affect their relationship with the exposure or outcome 3. IVs are only associated with outcome through exposure (no independent association). |
| 6 | **Statistical methods: main analysis** | Describe statistical methods and statistics used |  | Inverse variable weighted MR was performed for autism and the 8 co-occurring conditions. |
|  | a) | Describe how quantitative variables were handled in the analyses (i.e., scale, units, model) |  | - |
|  | b) | Describe how genetic variants were handled in the analyses and, if applicable, how their weights were selected |  | Only genetic variants from the GWAS summaries were used for both the exposure and outcome. |
|  | c) | Describe the MR estimator (e.g. two-stage least squares, Wald ratio) and related statistics. Detail the included covariates and, in case of two-sample MR, whether the same covariate set was used for adjustment in the two samples |  | Wald ratio estimates in a fixed-effect meta-analysis model were used to estimate the potential causal effect (IVW-MR). |
|  | d) | Explain how missing data were addressed |  | Variants with missing data were not included in the analyses. |
|  | e) | If applicable, indicate how multiple testing was addressed |  | MR-Egger analyses were performed to better consider the effect of multiple testing on the MR estimate. |
| 7 | **Assessment of assumptions** | Describe any methods or prior knowledge used to assess the assumptions or justify their validity |  | LD pruning was performed on the genetic variants by clumping the variants using PLINK. The possible effect of pleiotropy was explored using MR-Egger. It is important to note that the IV assumptions cannot fully be assessed using genetic variants from summary statistics. |
| 8 | **Sensitivity analyses and additional analyses** | Describe any sensitivity analyses or additional analyses performed (e.g. comparison of effect estimates from different approaches, independent replication, bias analytic techniques, validation of instruments, simulations) |  | MR-Egger was performed to gauge the heterogeneity and effect of pleiotropy. Potential causal directions were further explored using MR Steiger directionality testing. |
| 9 | **Software and pre-registration** |  |  |  |
|  | a) | Name statistical software and package(s), including version and settings used |  | MR analyses were performed using R-package TwoSampleMR (Hemani et al. 2018), version 0.5.10. R version 4.4.0 was used to perform the analyses. PLINK clumping of variants was performed using the ‘ieugwasr’ package and European population data from 1000 Genomes. |
|  | b) | State whether the study protocol and details were pre-registered (as well as when and where) |  | The study protocol was not pre-registered. |
|  | **RESULTS** |  |  |  |
| 10 | **Descriptive data** |  |  |  |
|  | a) | Report the numbers of individuals at each stage of included studies and reasons for exclusion. Consider use of a flow diagram |  | - |
|  | b) | Report summary statistics for phenotypic exposure(s), outcome(s), and other relevant variables (e.g. means, SDs, proportions) |  | Sources for summary GWAS data used are listed in S. Table 1. |
|  | c) | If the data sources include meta-analyses of previous studies, provide the assessments of heterogeneity across these studies |  | Not applicable |
|  | d) | For two-sample MR:  i.  Provide justification of the similarity of the genetic variant-exposure associations between the exposure and outcome samples  ii.  Provide information on the number of individuals who overlap between the exposure and outcome studies |  | 1. Significant genetic correlation thresholding was computed using LDSC to justify similarity. Variant associations with both exposure and outcome are obtained from large GWAS summary statistics 2. The proportion of ADHD among ASD cases in the iPSYCH cohort was 19.8%, and the proportion of ASD among ADHD cases was 16.1% (Mattheisen et al. 2022). As indicated and reference in manuscript, MTAG was ran to address multivariate sample overlaps. |
| 11 | **Main results** |  |  |  |
|  | a) | Report the associations between genetic variant and exposure, and between genetic variant and outcome, preferably on an interpretable scale |  | Associations between genetic variants with exposure and outcome are reported in the GWAS summary statistics. Complete list summary statistic sources in S. Table 1. |
|  | b) | Report MR estimates of the relationship between exposure and outcome, and the measures of uncertainty from the MR analysis, on an interpretable scale, such as odds ratio or relative risk per SD difference |  | MR estimates for the relationships between exposure traits and outcome are listed in S. Table 4. |
|  | c) | If relevant, consider translating estimates of relative risk into absolute risk for a meaningful time period |  | Not relevant |
|  | d) | Consider plots to visualize results (e.g. forest plot, scatterplot of associations between genetic variants and outcome versus between genetic variants and exposure) |  | Scatter plots for all IVW-MR analyses are available in the project GitHub under MR results (<https://github.com/jakelin212/mvasd_gwas>). |
| 12 | **Assessment of assumptions** |  |  |  |
|  | a) | Report the assessment of the validity of the assumptions |  | Mendelian randomization causal estimates are only valid if the IV assumptions are met. The selected IVs selected from the exposure trait summary statistics are known to be significantly associated with the exposure. Potential pleiotropy was assessed with MR-Egger testing which suggests some pleiotropy when compared to the IVW method. |
|  | b) | Report any additional statistics (e.g., assessments of heterogeneity across genetic variants, such as *I^2^*, Q statistic or E-value) |  | F1 scores were computed for all lead variants where their scores > 25 ( > 10 is considered strong). |
| 13 | **Sensitivity analyses and additional analyses** |  |  |  |
|  | a) | Report any sensitivity analyses to assess the robustness of the main results to violations of the assumptions |  | MR-Egger regression was performed to assess the effect of pleiotropy. |
|  | b) | Report results from other sensitivity analyses or additional analyses |  | - |
|  | c) | Report any assessment of direction of causal relationship (e.g., bidirectional MR) |  | Direction of the causal relationship was assessed using Steiger directionality testing. |
|  | d) | When relevant, report and compare with estimates from non-MR analyses |  | We performed colocalization analyses which provide strong evidence of shared genetic aetiology between autism and 3 of the co-occurring conditions (education attainment, schizophrenia and bipolar traits). |
|  | e) | Consider additional plots to visualize results (e.g., leave-one-out analyses) |  | - |
|  | **DISCUSSION** |  |  |  |
| 14 | **Key results** | Summarize key results with reference to study objectives | Mendelian Randomization | Aim of the study is to explore the relationship between autism and its co-occurring conditions. The results obtained from IVW MR -analyses suggest that the presence of a co-occurring condition significantly increases the probability of autism. |
| 15 | **Limitations** | Discuss limitations of the study, taking into account the validity of the IV assumptions, other sources of potential bias, and imprecision. Discuss both direction and magnitude of any potential bias and any efforts to address them |  | Estimates obtained from the performed MR analyses are valid only if the IV assumptions are met. In addition to IVW MR, MR-Egger was performed to address the possible effect of pleiotropy and to correct for multiple testing. Directionality of the association was further explored using Steiger directionality testing. |
| 16 | **Interpretation** |  |  |  |
|  | a) | Meaning: Give a cautious overall interpretation of results in the context of their limitations and in comparison with other studies |  | Autism has been linked to multiple co-occurring conditions in previous studies (Khachadourian et al. 2023; Romero et al. 2016). Our results confirm that all of the 8 co-occurring conditions tested are associated with autism. |
|  | b) | Mechanism: Discuss underlying biological mechanisms that could drive a potential causal relationship between the investigated exposure and the outcome, and whether the gene-environment equivalence assumption is reasonable. Use causal language carefully, clarifying that IV estimates may provide causal effects only under certain assumptions |  | Autism has previously been linked to multiple co-occurring conditions, such as ADHD, depression, anxiety, schizophrenia and learning difficulties. The concomitant development of these neurological conditions together with autism is assumed to result from complex interplay of genetics together with environment. From our MR analyses of the association of the 8 tested co-occurring conditions, all were found to significantly increase the probability of autism (lead SNP F1 > 25), which is in accordance with the assumption of shared genetic background of autism and the co-occurring conditions. Although it is important to note that the MR analysis results are only suggestive as the causal effect estimates can be provided only within the limits of the IV assumptions. |
|  | c) | Clinical relevance: Discuss whether the results have clinical or public policy relevance, and to what extent they inform effect sizes of possible interventions |  | Not applicable |
| 17 | **Generalizability** | Discuss the generalizability of the study results (a) to other populations, (b) across other exposure periods/timings, and (c) across other levels of exposure |  | This study used genome-wide summary statistics of mainly individuals with European ancestry and may not be directly applicable to more diverse populations. |
|  | **OTHER INFORMATION** |  |  |  |
| 18 | **Funding** | Describe sources of funding and the role of funders in the present study and, if applicable, sources of funding for the databases and original study or studies on which the present study is based | 18 | The work was supported by the European Commission Horizon 2020 programme (grant no. 825033). We are sincerely grateful to the entire GEMMA team and in particular to the families participating in the project. |
| 19 | **Data and data sharing** | Provide the data used to perform all analyses or report where and how the data can be accessed, and reference these sources in the article. Provide the statistical code needed to reproduce the results in the article, or report whether the code is publicly accessible and if so, where | 18 | Summary source stated in S Table 1 and R markdown provided in GitHub: <https://github.com/jakelin212/mvasd_gwas>. |
| 20 | **Conflicts of Interest** | All authors should declare all potential conflicts of interest | 18 | All authors declared that we do not have any potential conflicts of interest. |

This checklist is copyrighted by the Equator Network under the Creative Commons Attribution 3.0 Unported (CC BY 3.0) license.

1. Skrivankova VW, Richmond RC, Woolf BAR, Yarmolinsky J, Davies NM, Swanson SA, et al. Strengthening the Reporting of Observational Studies in Epidemiology using Mendelian Randomization (STROBE-MR) Statement. JAMA. 2021;under review.

2. Skrivankova VW, Richmond RC, Woolf BAR, Davies NM, Swanson SA, VanderWeele TJ, et al. Strengthening the Reporting of Observational Studies in Epidemiology using Mendelian Randomisation (STROBE-MR): Explanation and Elaboration. BMJ. 2021;375:n2233.
