# Supplementary material for: Exploring autism spectrum disorder and co-occurring trait associations to elucidate multivariate genetic mechanisms and insights
Source: BMC Psychiatry. 2024 Dec 18;24:934. doi: 10.1186/s12888-024-06392-w (PMC11658126; doi:10.1186/s12888-024-06392-w)
Supplement: Supplementary file 2 — Supplementary Material 2. [file 12888_2024_6392_MOESM2_ESM.pdf]

The GEMMA Study Group:

Institute	Researchers
EBRIS: European Biomedical Research Institute Salerno, Italy	Alessio Fasano<br>Corrado Vecchi<br>Giorgia Venutolo<br>Federica Farina<br>Dario Siniscalco
NUT: Danone Research & Innovation	Guus Roeselers<br>Kumar Himanshu<br>Ioannis Kostopoulos
BMS: Bio-Modeling Systems, France	Thanos Beoupoulos<br>Francois Iris<br>Manuel Gea
EUF: Euformatics Oy, Finland	Christophe Roos
THE Theoreo SRL, Italy	Martina Lombardi<br>Jacopo Troisi
NUIG: National University of Ireland Galway, Ireland	Lokesh Joshi<br>Geraldine Leader<br>Sally Whelan<br>Stephen Cunningham
ASL: Azienda Sanitaria Locale Salerno, Italy	Giulio Corrivetti
MGH: Massachussets General Hospital for Children Harvard Medical School, USA	Emma Breton Guerette<br>Sarah Kadzielski
CNR: National Council of Research Institute of Biomedical Technologies, Italy	Alessandra Mezzelani<br>Ettore Mosca<br>Matteo Gnocchi<br>Marco Moscatelli
INRAE: French National Institute for Agriculture, Food and the Environment, France	Sylvie Rabot<br>Emmanuelle Le Chatelier<br>Léa Roussin<br>Laurent Naudon<br>Christian Morabito<br>Benoit Quinquis<br>Alexandre Famechon<br>Elise Maximin<br>Magali Monnoye
Neuroendocrine, Endocrine and Germinal Differentiation and Communication Laboratory, Inserm UMR1239, University of Rouen Normandie, 76000, Rouen, France	Serguei Fetissov
UU: Utrecht University, Netherlands	Aletta D Kraneveld<br>Lucia Peralta Marzal<br>Paula Perez-Pardo<br>Naika Prince
TAU: Tampere University, Finland	Reija Autio<br>Jake Lin<br>Karoliina Salenius<br>Sini Thusberg<br>Niina Väljä<br>Matti Nykter

Division of Digestive Diseases, Department of Metabolism, Digestion and Reproduction, Faculty of Medicine, Hammersmith Campus, Imperial College London, W12 0NN, U.K.<br>And<br>The Australian National Phenome Center and Centre for Computational and Systems Medicine, Harry Perkins Institute, Murdoch University, WA 6150, Australia	Elaine Holmes
JHU: Johns Hopkins University, USA	Christine Ladd-Acosta<br>Jackie Bidinger<br>M. Daniele Fallin
